# Patients with inflammatory bowel disease have a higher chance of developing periodontitis: A systematic review and meta-analysis

**DOI:** 10.3389/fmed.2022.1020126

**Published:** 2022-11-08

**Authors:** Zsuzsanna Domokos, Eszter Uhrin, Bence Szabó, Márk László Czumbel, Fanni Dembrovszky, Beáta Kerémi, Gábor Varga, Péter Hegyi, Péter Hermann, Orsolya Németh

**Affiliations:** ^1^Department of Community Dentistry, Semmelweis University, Budapest, Hungary; ^2^Centre for Translational Medicine, Semmelweis University, Budapest, Hungary; ^3^Department of Periodontology, Semmelweis University, Budapest, Hungary; ^4^Institute for Translational Medicine, Szentágothai Research Centre, Medical School, University of Pécs, Pécs, Hungary; ^5^Department of Restorative Dentistry and Endodontics, Semmelweis University, Budapest, Hungary; ^6^Department of Oral Biology, Semmelweis University, Budapest, Hungary; ^7^Division of Pancreatic Diseases, Heart and Vascular Centre, Semmelweis University, Budapest, Hungary; ^8^Department of Prosthodontics, Semmelweis University, Budapest, Hungary

**Keywords:** periodontal disease, inflammatory bowel diseases (IBD), Crohn’s diseases, ulcerative colitis, multifactorial diseases, meta–analysis, systematic review, oral health

## Abstract

**Background and objective:**

Periodontitis affects up to one billion people worldwide, and has been proven to be associated with several systemic inflammatory conditions. This study investigates the specific relationship between two multifactorial diseases: Inflammatory bowel disease (IBD) and periodontitis. To thoroughly explore this issue, we investigated separately whether IBD patients have a higher chance of developing periodontitis, and equally, whether patients with periodontitis have a higher chance of developing IBD.

**Methods:**

The systematic search was performed in three databases: MEDLINE, Cochrane Trials, and Embase, up to 26 October 2021. The protocol was registered in PROSPERO. All eligible studies investigating the association between IBD and periodontitis from either direction were included. The Newcastle-Ottawa Scale was used to assess the risk of bias. As a primary outcome, we investigated the prevalence of IBD and periodontitis, and calculated the odds ratio (OR). Our secondary outcomes involved comparing the clinical periodontal outcomes of IBD patients to those of IBD-free patients.

**Results:**

The systematic search resulted in 1,715 records, 14 of which were eligible for qualitative synthesis and 8 for quantitative synthesis. On the basis of the results of the primary outcome, IBD diagnosis was associated with significantly higher odds of periodontitis: OR = 2.65 (CI: 2.09-3.36, *I*^2^ = 0 (CI: 0-0.75)). For subgroup analysis, we investigated separately the odds in Crohn’s disease (CD) patients: OR = 2.22 (CI: 1.49-3.31, *I*^2^ = 0.05 (CI: 0-0.76)) and in ulcerative colitis (UC) patients: OR = 3.52 (CI: 2.56 to 4.83, *I*^2^ = 0 (CI: 0-0.75)); the odds were significantly higher in all cases. Two studies investigated whether patients with periodontitis were more susceptible to IBD, and both found that periodontitis was significantly associated with the risk of subsequent UC, but not with subsequent CD. However, more studies are needed to prove an association.

**Conclusion:**

Our analysis confirmed that IBD patients have a higher chance of developing periodontitis, and are a higher risk population in dentistry. Both dentists and gastroenterologists should be aware of this relationship and should emphasize the importance of prevention even more than in the healthy population.

**Systematic review registration:**

[https://www.crd.york.ac.uk/prospero/], identifier [CRD42021286161].

## Key points

(1) IBD patients have a higher chance of developing periodontitis; they are therefore considered a risk population in dentistry.

(2) There are several pathological pathways and common risk factors shared between the two diseases, which can contribute to the link between dental and gastroenterological diseases.

## Introduction

Periodontal disease is reported to be one of the most common oral conditions in the world. According to the Global Burden of Disease Study 2019, severe periodontal diseases are estimated to affect 14% of the adult population, reaching more than one billion cases worldwide (1). Periodontal diseases are chronic inflammatory, destructive processes involving the periodontium – the supportive apparatus surrounding a tooth (2). In an advanced stage, it causes alveolar bone resorption, and is the major cause of tooth loss (3).

Periodontitis is a multifactorial disease. It is known that poor oral hygiene and dental biofilm accumulation are the major causes of periodontitis, but genetic predisposition, other environmental and systemic conditions, and a pathologic immune-inflammatory response also play key roles in the clinical manifestation (4). There are several systemic diseases that are known to be associated with the development of periodontitis; diabetes mellitus (5) hematological disorders, and immunodeficiencies (6) are the best-known systemic diseases that render patients susceptible to periodontitis.

Periodontitis causes serious oral health complications; however, it is also known to affect systemic health. Periodontal pathogenic bacteria can enter the circulation through the infected pockets by dissemination and can cause various serious complications (7). It has been shown to be capable of contributing to adverse pregnancy outcomes (8), coronary heart disease (9, 10), stroke (10), rheumatoid arthritis (11), neurological complications (12), respiratory disorders (13), and cancers (14). According to recent studies, increasing attention has been paid to the observation that inflammatory bowel diseases also appear to be associated with periodontitis (15–17).

Inflammatory bowel diseases (IBD) are chronic inflammatory intestinal diseases with two forms: Crohn’s disease (CD) and ulcerative colitis (UC). The growing incidence of these diseases might be associated with modern lifestyles (18). The highest annual incidence of CD is in Europe, at 12.7 per 100,000 person-years; the figure for UC in Europe is 24.3 per 100,000 person-years (19). CD can cause symptoms through the entire gastrointestinal tract, and causes skip lesions and transmural inflammation (20), while UC affects only the rectum and the colon, and causes continuous mucosal inflammation (21). Family accumulation can be observed in both cases (18) and, similarly to periodontitis, abnormal immune-inflammatory responses also play a key role in the pathogenesis. Relapses and remissions follow each other throughout the life of the patient, but the actual causes of a relapse are partly unknown (22). However, systemic inflammation can contribute to a relapse (23). It is also known that IBD has extra-intestinal manifestations as well. These can cause skin lesions (erythema nodosum and pyoderma gangrenosum), joint disorders (peripheral and axial arthropathies), eye disorders (episcleritis and uveitis), and hepatobiliary disorders (primary sclerosing cholangitis) (24). It has also been observed that IBD can have several oral manifestations and cause oral symptoms, like cobblestoning, mucosal tags, aphthous ulcerations, and pyostomatitis vegetans (25, 26).

Our hypothesis is that both multifactorial, immune-inflammatory diseases, i.e., IBD and periodontitis, are associated with one another. As the pathogenesis of neither disease is fully understood, investigating the association and the common processes between them could help to understand the pathogenesis of both diseases and to improve treatment options. If there is a bidirectional association between them, the treatment of IBD patients should always be a multidisciplinary activity involving both dentists and gastroenterologists.

Several earlier original studies (15–17, 27–37) and also some previous meta-analyses (38–41) have focused on investigating the association between IBD and periodontitis, and they all found a positive association. However, these studies had several limitations. They only investigated whether IBD might be a risk factor for periodontitis, but they failed to investigate whether periodontitis might be a risk factor for IBD. Also, it should be noted that one study (40) compared studies targeting different exposed groups and control groups, introducing bias. We collected studies separately to investigate the association in both directions and followed a rigorous methodology throughout the process of checking previous results. Additionally, we were able to include recent studies that had been unavailable to the previous meta-analyses.

The aim of our study was to investigate the bidirectional association between periodontitis and inflammatory bowel diseases through a systematic review and meta-analysis. We set this goal to improve the precision of the meta-analysis, thus surpassing the relevance of previous results.

## Materials and methods

### Protocol and registration

We reported our systematic review and meta-analysis based on the PRISMA 2020 guideline (42), following the Cochrane Handbook (43). The protocol of the study was registered in advance in the International Prospective Register of Systematic Reviews (PROSPERO) (registration number: CRD42021286161).

### Eligibility criteria

All studies investigating the association between IBD and periodontitis were considered eligible for our systematic review and meta-analysis. We defined two questions and formed two PECO (patient/population-exposure-control-outcome) frameworks, including studies using different exposed and control groups, in order to investigate the association from both directions (Supplementary Tables 1, 2).

Question 1 - PECO 1: Studies investigating clinical periodontal outcomes and the presence of periodontitis in patients with IBD and non-IBD controls: Population (P): human subjects, regardless of age or sex (exclusion: edentulous patients); Exposure (E): diagnosis of IBD (including CD or UC) regardless of type of IBD, treatment for IBD, or time of IBD diagnosis; Control (C): absence of IBD; main Outcome (O): prevalence of periodontitis; secondary outcome: any clinical periodontal parameters examined in the study (Probing Pocket Depth (PPD), Gingival Recession (GR), Clinical Attachment Loss (CAL), Bleeding On Probing (BOP), Plaque Index (PI), Gingival Index (GI), Community Periodontal Index of Treatment Needs (CPITN), etc.)

Question 2 - PECO 2: Studies providing data about the presence of IBD in patients with periodontitis and patients with healthy periodontium. P: human subjects, regardless of age or sex; E: diagnosis of periodontitis accompanied by the definition of the disease as given by the authors; C: absence of periodontitis; O: prevalence of IBD (either CD or UC).

Case reports, case series, animal studies, *in vitro* studies, review articles, abstracts, posters, letters, and editorials were excluded from the selection. English language articles were screened.

### Information sources and search strategy

Our systematic search was conducted on 26 October 2021 in three electronic databases: MEDLINE (via PubMed), EMBASE, and the Central Cochrane Register of Controlled Trials (CENTRAL). No filters were applied. We manually searched for additional eligible studies in the reference lists of review articles.

During the systematic search the following search key was used:

(periodontitis OR chronic periodontitis OR periodontal OR periodontal disease) AND (inflammatory bowel disease OR Crohn* OR ulcerative colitis OR uc OR cd OR ibd) (Supplementary Table 3).

### Selection process

After the systematic search, the references were imported into a reference management software (EndNote X9, Clarivate Analytics) and screened for duplicates, which were removed automatically and by manual checking. After duplicate removal, the selection was performed independently by two review authors (ZD and EU), first by title and abstract. Then the full text of articles meeting our exclusion criteria were screened. Cohen’s kappa coefficient was calculated to measure interrater reliability during the selection process (44). Disagreements were resolved by a third author (ON).

### Data collection process and data items

Data were extracted independently in a pre-defined Excel (Microsoft Corporation, Redmond, Washington, United States) data sheet by two review authors (ZD and EU). In the case of missing data, we contacted the corresponding author. The following data were extracted: first author, the year of publication, study design, country, study size, type of IBD, demographic data, including the mean age of each group, as well as extra data, such as smoking habits, comorbidities, drugs used for IBD, and the applied definition of periodontitis.

The primary outcome, which was defined as the total number of patients and those with the event of interest was extracted from each study. For PECO 1 the event of interest was periodontitis and for PECO 2 the event of interest was IBD in each group separately for OR. If the number of patients with the event of interest could not be extracted but the OR was available, the counted OR values were used.

Secondary outcomes were defined as the value of different clinical periodontal outcomes (CAL, PPD, BOP, PI, GI, etc.) measured in the IBD and IBD-free groups. For continuous outcomes the difference between the mean of the IBD and the healthy population was used for the effect size measure.

### Study risk of bias assessment

For case-control and cohort studies, the Newcastle-Ottawa Scale was used to assess the quality of the included studies (45). The quality assessment was performed independently by two authors (ZD, EU). Discrepancies were resolved by a third review author (ON).

A study is judged from three main perspectives: the selection of study groups, the comparability of groups, and the ascertainment of the exposure. The lowest quality study receives 0 stars, and the highest receives 9 stars. Studies under 5 stars are considered low; above 5 they are considered moderate or high quality studies (45).

### Synthesis methods and effect measures

For continuous outcomes, the difference between the mean of the IBD and the healthy population was used for the effect size measure. To calculate the pooled difference, the sample size, mean, and corresponding standard deviation were extracted from each study. For categorical outcomes, the odds ratio (OR) with a 95% confidence interval (CI) was used for the effect size measure. To calculate the OR, the total number of patients and those with the event of interest (in each group separately for OR) was extracted from each study. If available, the OR values were used if the patient quantity with the event of interest could not be extricated.

For continuous outcomes, the difference between the mean of the IBD and the healthy population was used for the effect size measure. To calculate the pooled difference, the sample size, mean, and corresponding standard deviation were extracted from each study. As we anticipated considerable between-study heterogeneity, a random-effects model was used to pool effect sizes. Pooled OR was calculated using the Mantel-Haenszel method (46–48). The exact Mantel-Haenszel method (without continuity correction) was used to handle zero cell counts (49, 50), and the inverse variance weighting method was used to calculate the pooled mean difference. For the outcomes where the study number was over 5, a Hartung-Knapp adjustment was used (51, 52); we did not apply this adjustment below 5. To estimate the heterogeneity, the variance measure τ^2^ was used. For continuous outcomes the restricted maximum-likelihood estimator was applied, and for OR measures the Paule-Mandel method (53) recommended by Veroniki et al. (54) was applied to estimate the variance with the Q profile method for the confidence interval. Additionally, between-study heterogeneity was described by means of Cochrane’s *Q* test and Higgins and Thompson’s I^2^ statistics (55). Forest plots were used to graphically summarize the results. For mean difference, the effect size measuring the confidence interval of an individual study was calculated based on the *t*-distribution. Where applicable, we reported the prediction intervals (i.e., the expected range of effects of future studies) of results following the recommendations of IntHout et al. (52). Outlier and influence analyses were carried out following the recommendations of Harrer et al. (56) and Viechtbauer and Cheung (57). Publication bias was planned to be assessed with Egger’s test (at a significance level of 10% due to the small study number) using the Harbord method for binary outcome measures (58) and classical Egger’s method for continuous outcomes to calculate the test statistic (59). However, the number of studies included was below 10; therefore, Egger’s test would have lacked the statistical power to detect bias, or could give a false “positive” result, which is why these results should not be evaluated.

All statistical analyses were made with R [v4.1.1] using the meta [5.0.0] package.

### Additional analysis

We assessed the certainty of evidence by using the Grading of Recommendations Assessment, Development, and Evaluation (GRADE) for the outcomes measured in the meta-analysis (60). On the basis of the assessed domains, the overall quality of evidence was classified for each outcome as high, moderate, low, or very low. Additionally, a subgroup analysis was conducted based on IBD type. Publication bias could not be analyzed with high certainty, as the number of available studies did not reach the minimum required sample size.

## Results

### Search and selection

**FIGURE 1:**
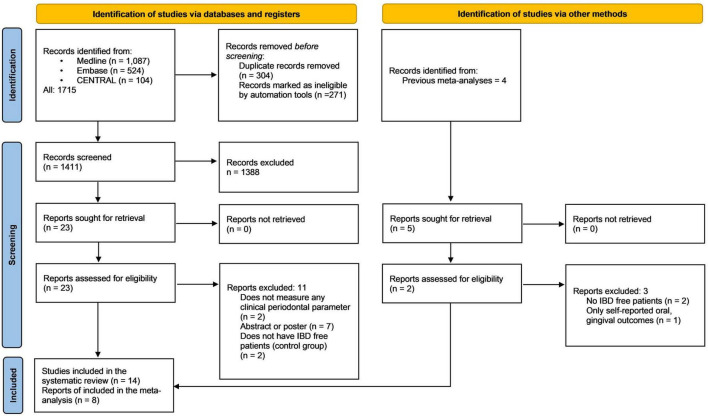
Preferred reporting items for systematic reviews and meta-analyses (PRISMA) flowchart of study selection.

In all, 1,715 records were identified by our search query; this was reduced to 1,411 records after duplicate removal. These 1,411 studies were screened by title and abstract (Cohen’s Kappa: 0.85), and the full texts of 23 articles were screened (Cohen’s Kappa: 0.91). We manually searched the reference lists of previous review articles. Five additional articles were screened, and two met our inclusion criteria. Through the selection process, fourteen articles were enrolled in the qualitative synthesis (15–17, 27–37), eight were included in the quantitative synthesis (15–17, 27, 29, 30, 36, 37), and six of them could be used to answer the main outcome (16, 17, 27, 30, 36, 37). The flowchart of the selection can be seen in Figure 1.

#### Excluded studies

According to our PECO 1, we found seven eligible articles. However, we excluded one study from the final statistical evaluation because it investigated only Crohn’s disease patients instead of both IBD forms, thereby introducing bias (28) (Supplementary Figure 1).

There were several studies that contained information on the periodontal status of IBD patients and the control group. However, the prevalence of periodontitis could not be extracted from these studies, so they could not be utilized in the statistical evaluation of the primary outcome (15, 29, 32, 35).

According to our PECO 2 we found 2 eligible studies (31, 33).

### Basic characteristics of included studies

Case-control and cohort studies were included in our systematic review and meta-analysis (Table 1) (15–17, 27–35, 37).

**TABLE 1:** Basic characteristics of included studies.

Study (year)	Study design	Country	Patients (mean age)	Smoking habitual (smoker; non-smoker; former smoker)	Drugs used for IBD	Comorbidities	Definition of periodontitis
Grössner-Schreiber et al. (29)	Case-control	Germany	IBD: 62 CD: 46 UC: 16 Control: 59	IBD: 25 (40%); 34 (55%); 3 (5%) CD: 24; x; x UC: 1; x; x Control: 24 (41%); 29 (49%); 6 (10%)	CS (*n* = 20) IS (e.g., AZA, MTX) (*n* = 24) ASA (*n* = 39) Anti-TNF (*n* = 13) AB (*n* = 12) Mono- or combined therapy	No data	Not defined
Zervou et al. (36)	Case-control	Greece	IBD: 30 (40) CD: 15 UC: 15 Control: 47 (43)	No data	Mesalazine (*n* = 22) AZA (*n* = 2)	No data	Not defined
Brito et al. (27)	Case-control	Brazil	IBD: 179 CD: 99 (39) UC: 80 (43) Control: 74 (40)	IBD: 19; 101; 59 CD: 12 (12.1%); 63 (63.6%); 24 (24.3%) UC: 7 (8.7%); 38 (47.5%); 35 (43.8%) Control: 9 (12.2%); 57 (77.0%); 8 (10.8%)	No medication (*n* = 9) ASA (*n* = 78) IM (*n* = 25) ASA + IM (*n* = 26) ASA + CS (*n* = 17) IM + CS (*n* = 11) ASA + IM + CS (*n* = 13) Anti-TNF (*n* = 10) Metronidazol (*n* = 5) Ciprofloxacin (*n* = 4)	IBD: HT, DM, EIM Control: no data	Having CAL ≥ 3mm in at least four sites in different teeth.
Habashneh et al. (30)	Case-control	Jordan	IBD: 160 CD: 59 UC: 101 Control: 100	IBD: 48; 78; 34 CD: 31 (52.5%); 23 (39%); 5 (8.5%) UC: 17 (16.8%); 55 (54.5%); 29 (28.7%) Control: 49 (49%); 44 (44%); 7 (7%)	No data	HT	Presence of four or more teeth with one or more sites with probing pocket depth ≥ 4mm and clinical attachment level ≥ 3mm.
Slebioda et al. (34)	Case-control	Poland	IBD: 95 CD: 70 (37,4) UC: 25 (37,2) Control: 70 (31,6)	No data	No data	No data	No data
Vavricka et al. (37)	Case-control	Germany	IBD: 13 (40.6) CD: 69 (39.6) UC: 44 (42.3) Control: 113 (41.7)	IBD: 23 (20.4%); 48 (42.5%); 42 (37.2%) CD: 21 (30.4%); 25 (36.2%); 3 (4.3%) UC: 2 (4.5%); 23 (52.3%); 19 (43.2%) Control: 21 (18.6%); 71 (63.8%); 21 (18.6%)	CS (*n* = 24) AS (*n* = 37) Thiopurines (AZA & 6-MP; *n* = 25) MTX (*n* = 5) Cyclosporine, Tacrolimus (*n* = 3, 3) Anti-TNF (IFX, ADA, CZP *n* = 45) Probiotics (*n* = 8) NSAID (*n* = 3)	IBD: EIM, alcohol Control: alcohol	Defined as LA-PPD ≥ 5mm and/or BOP
Koutschristou et al. (32)	Case-control	Greece	IBD: 55 (12.27) CD: 36 UC: 19 Control: 55 (all < 18)	No data	AS CS Anti-TNF IM (*n* = 28) Combination of 2 or 3 drugs (*n* = 55)	No data	No data
Chi et al. (28)	Cohort study	Taiwan	IBD: 6,657 CD: 6,657 UC: 0 Control: 26,628	No data	ASA (*n* = 162) AZA (*n* = 24) Other IS (*n* = 136) CS (*n* = 5766) Non-IBD related medications control group takes medication too	IBD: HT, HL, CHD, DM, HF, RD Control: HT, HL, CHD, DM, HF, RD	ICD-9-CM code: 523.3 or 523.4 (bi-annual checkups periodontal examination, probing of the sulcus and radiographs a depth of 3 mm or greater is considered to support a diagnosis of peri-odontal disease)
Schmidt et al. (15)	Cross-sectional study, case-control	Germany	IBD: 59 (49.8) CD: 30 (49.6) UC: 39 (50.0) Control: 59 (51.3)	IBD: 0 CD: 14 (48%) UC: 0 Control: 0	AS CS Anti-TNF IM single or combination	No data	No data
Yu et al. (16)	Retrospective cohort study	Taiwan	IBD: 27 (38) CD: 7 UC: 20 Control: 108 (36,3)	No data	no data	No data	ICD-9-CM diagnosis code (ICD-9-CM codes 523.3, 523.4, and 523.5). ICD-9 procedure code 9,654, 2,431, and 2,439 were also defined
Zhang et al. (17)	Case-control	China	IBD: 389 CD: 265 (29) UC: 124 (39) Control: 265 (26)	IBD: 35; 295; 59 CD: 21 (7.9%); 208 (78.5%); 36 (13.6%) UC: 14 (11.3%); 87 (70.2%); 23 (18.5%) Control: 22 (8.3%); 226 (85.3%); 17 (6.4%)	ASA (*n* = 94) CS (*n* = 28) IS (*n* = 133) Biologicial therapy (*n* = 113) Untreated (*n* = 21)	No data	≥2 interproximal sites with CAL ≥3 mm, and ≥2 interproximal sites with PD ≥4 mm (not on the same tooth), or ≥ 1 site with PD ≥ 5 mm
Tan et al. (35)	Case-control	Netherlands	IBD: 229 (51) CD: 148 UC: 80 + 1 Undetermined Control: 229 (51)	IBD: 53 CD: UC: Control: 72	CS (*n* = 36) Biologicials (*n* = 27) IS (*n* = 25) ASA (*n* = 59)	IBD: DM, alcohol Control: DM, alcohol	No data

IBD, inflammatory bowel disease; CD, Crohn’s disease; UC, ulcerative colitis; CS, corticosteroids; ASA, aminosalicilate; IS, immunosuppressants; IM, immunmodulators; IM, azathioprine; IM, methotrexate; 6-MP, 6-mercaptopurine; anti-TNF, anti-tumor necrosis factor; AB, antibiotics; IFX, infliximab; ADA, adalimumab; CZP, certolizumab pegol; NSAID, non-steroidal anti-inflammatory drugs; HT, hypertension; DM, diabetes mellitus; EIM, extraintestinal manifestations of IBD; HL, hyperlipidaemia; CHD, coronary heart disease; HF, heart failure; RD, renal disease; CAL, clinical attachment loss; LA-PPD, largest-periodontal probing depth; BOP, bleeding on probing; ICD-9-CM, international classification of diseases, ninth version, clinical modification; PD, pocket depth.

### Risk of bias assessment

The results of the risk of bias assessment are presented in Supplementary Tables 4, 5. As all studies included attained at least 6 stars, they are considered to have low or moderate bias risk levels. These findings suggest that the included studies have a moderate or high quality of methodology. The selection of controls caused bias in most cases. Due to the small number of studies, a formal assessment of reporting bias was not possible.

### Results of individual studies and synthesis

The basic characteristics of studies included in this work are presented in Table 1, and the results are further evaluated in the discussion. Supplementary Tables 7–10 contain the detailed data used for the statistical evaluation.

### Association between inflammatory bowel disease and periodontitis

**FIGURE 2:**
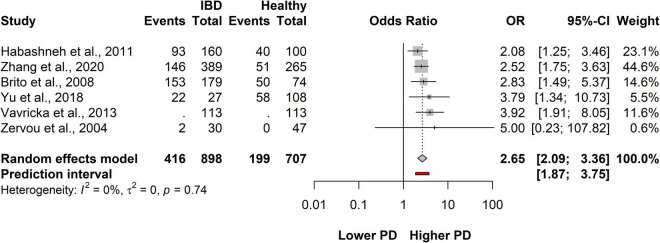
Forest plot showing the odds of developing periodontitis in inflammatory bowel disease (IBD) and IBD-free group.

From six articles, total 1,605 patients – out of which 898 were IBD and 707 were healthy – were used to evaluate the association between IBD and PD (16, 17, 27, 30, 36, 37) (Figure 2). On average, the OR (the pooled effect size) of having periodontitis (PD) was 2.65, (CI: 2.09-3.36) which represents a statistical difference between the investigated groups. These results suggest that the odds of having PD in the IBD population are higher than in the healthy population. The between-study heterogeneity expressed as an I^2^ value was 0 (95% CI: 0 - 0.75). The variance of true effects (τ^2^) was 0, and the standard deviation of true effects (τ^2^) was 0. The prediction interval was 1.87-3.75.

These results suggest that patients with IBD have a higher chance of developing periodontitis than the IBD-free population (Figure 2 and Supplementary Figure 2).

All studies reported both on CD patients and UC patients. By subgroup analysis, we statistically evaluated these diseases separately compared to the IBD-free population.

### Association between Crohn’s disease and periodontitis

**FIGURE 3:**
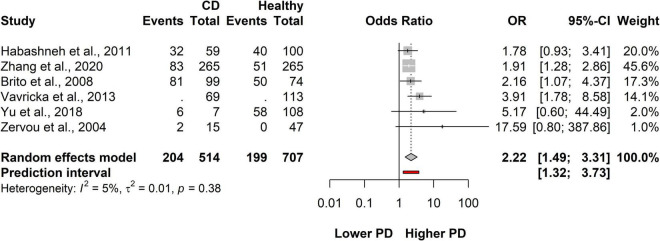
Forest plot showing the association between Crohn’s disease (CD) and periodontitis.

A total of six studies covering a total of 1,605 patients, 514 were classified as CD and 707 were healthy population. On average, the OR (the pooled effect size) of having PD was 2.22 (CI: 1.49-3.31). The between-study heterogeneity expressed as an I^2^ value was 0.05 (95% CI: 0-0.76). We can conclude that the odds of having PD in the CD population are higher than those of the healthy population (Figure 3 and Supplementary Figure 3).

### Association between ulcerative colitis and periodontitis

**FIGURE 4:**
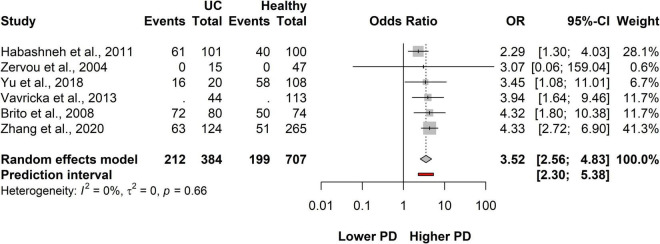
Forest plot showing the association between ulcerative colitis (UC) and periodontitis.

A total of six studies covering a total of 1,605 patients in all, 384 were classified as having UC and 707 were healthy population. On average, the OR (the pooled effect size) of having PD was 3.52 (CI: 2.56-4.83). The between-study heterogeneity expressed as an I^2^ value was 0 (95% CI: 0-0.75). We can conclude that the odds of having PD in the UC population are higher than those in the healthy population (Figure 4 and Supplementary Figure 4). The OR is the highest in the UC population compared to controls.

According to our results, it could be concluded that both CD and UC separately are significantly associated with developing periodontitis. Further evaluation is detailed in Supplementary Appendix 1.

### Risk of developing inflammatory bowel disease in patients with periodontitis compared to patients with a healthy periodontium

We collected studies separately for PECO 2, which resulted in two studies only (31, 33). The examined population in the studies was huge (6,646 in total were classified with CD, 6,108 were classified as UC patients, and 10,085,738 were the healthy population, based on International Classification of Diseases (ICD)-codes from health insurance databases). However, due to the low number of studies, only tendencies could be examined, and the statistical results are not reliable.

**FIGURE 5:**
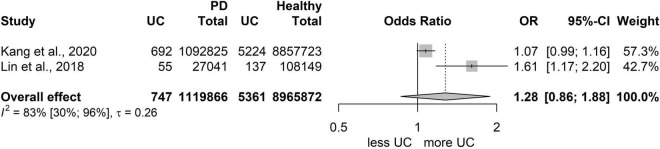
Risk for developing ulcerative colitis (UC) in patients with periodontitis.

**FIGURE 6:**
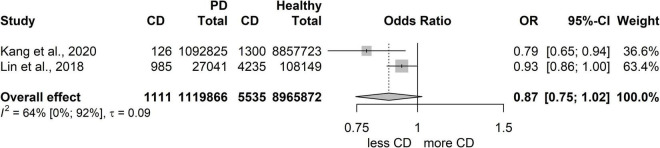
Risk for developing Crohn’s disease (CD) in patients with periodontitis.

However, both studies (31, 33) reached the conclusion that periodontitis was significantly associated with the risk of subsequent UC, but not with subsequent CD. Kang et al. and Lin et al. similarly found that the risk of UC in periodontitis patients was significantly higher than in patients with healthy periodontium (Kang: aHR: 1.091, 95% CI: 1.008-1.182; Lin: aHR: 1.56, 95% CI: 1.13-2.15), but not the risk of CD (Kang: aHR: 0.879, 95% CI: 0.731-1.057; Lin: aHR:0.99, 95% CI: 0.92-1.06). However, more studies are needed for a reliable statistical analysis; these results should be treated with some caution (Figures 5, 6).

### Secondary outcomes

As secondary outcomes, we planned to examine the different clinical periodontal parameters used in the eligible studies. However, due to the varied methods used to measure the periodontal parameters by different investigators, only a very low number of studies [5 PPD (15, 17, 27, 29, 30), and 3 in CAL (15, 27, 30)] which showed a high level of heterogeneity could be used for the statistical evaluation. These studies matched our PECO 1. Although the difference between the PPD results in the IBD group and the IBD-free group showed statistically significant values, the observed difference was actually not clinically relevant. Therefore, these results should be considered cautiously. In the case of CAL, the difference did not reach a statistically significant level (Supplementary Figures 5–9). The statistical evaluation of the other clinical parameters (GI, BOP, GI etc.) was not applicable due to the low number of studies using the same measuring method.

### Certainty of evidence: Grading of recommendations assessment, development, and evaluation

The overall quality of evidence in all three outcomes was found to be moderate, but the only reason for not classifying them as high was the study design, as the use of RCTs was not possible in our meta-analysis. We found no other reasons to downgrade the quality of evidence (Supplementary Table 6).

## Discussion

### Summary of main findings

In this systematic review and meta-analysis, we investigated the association between IBD (both CD and UC) and periodontitis. Our aim was to investigate the association from both directions in order to discover whether there was a two-way association between these diseases, and to overcome the limitations of previous meta-analyses.

Our results confirm that patients with IBD have a significantly higher chance of periodontitis (OR: 2.65). This is true for both CD (OR: 2.22) and UC (OR: 3.52), which were investigated separately. Therefore, it can be concluded that either type of IBD is associated with periodontitis.

The previously published meta-analyses on the topic concluded a higher risk of developing periodontitis in patients with IBD, and our finding show that the statements were correct (38–41). Our results show a weaker association between the diseases than the first meta-analysis on the topic (39), but new studies have been published since the latter was performed, and these are included in our results (16, 17). Notably, our results show a stronger association than the result of the latest meta-analysis on the topic (38).

### Summary of the investigated secondary outcomes

The results of the investigation of secondary outcomes showed that IBD patients have deeper mean PPD and CAL values than the IBD-free population. These results of the difference in PPD values show a lower difference between the groups than the results of the only meta-analysis to investigate this data (39). However, none of the results are clinically relevant.

### Summary of the investigated population in the studies

Various populations are covered by the studies, including European, Asian, and South American. According to the mean patient age, the population investigated was mostly middle-aged, except in one case where children were investigated (32). Most of the studies considered smoking, previously smoking, as well as and non-smoking patients although smoking may worsen periodontal outcomes. As the data on periodontal status with different subgroups based on smoking habit were missing, we could not perform an analysis that compared only IBD and IBD-free patients sharing the same smoking habit. Moreover, as for the medications used for IBD, the IBD population was very heterogeneous. Medication also is also known to affect the results, as does the coexistence of other systemic diseases. However, in almost all cases, having a serious systemic disease was an exclusion criterion, so no serious comorbidities could be noted. Additionally, the definition of periodontitis used by the investigators differed to some extent. We always used the definition employed by each group of investigators.

### Summary of the results of individual studies

We could not include the study that investigated only CD patients into the statistical evaluation, but it should be noted that Chi et al. carried out a longitudinal study to determine whether there was a causative link between the two diseases. They identified an increased risk of subsequent periodontitis among patients with CD (28). There were several studies that did not provide information about the prevalence of periodontitis among the exposed and the control populations; however, they did compare different periodontal outcomes (15, 29, 32, 35). Grössner-Schreiber et al. found that the mean PPD was deeper in the IBD population and observed that IBD patients had more sites with CAL of at least 5 mm (29). The results were similar in the study by Schmidt et al. (15); they classified periodontitis into healthy/mild, moderate, and severe cases, and found that IBD patients had more severe periodontitis, as their CAL results were higher. They were the first team to measure the active matrixmetalloproteinase-8 (aMMP-8) level of the gingival crevicular fluid (GCF) in the IBD and the control populations. Salivary aMMP-8 is higher in patients with periodontitis than in patients with healthy periodontium, and it is associated with periodontal destruction (61). They found a general increase in the GCF levels of aMMP-8 in the IBD group. In CD populations, aMMP-8 was associated with an increase in periodontitis severity (15).

There were studies that compared the CPITN Index results of the groups; Slebioda et al. found that IBD population had significantly higher CPITN index results than the healthy population (34). A Dutch team (Tan et al.) used a similar index, namely the Dutch Periodontal Screening Index (DPSI). Interestingly, the DPSI results between the IBD and control groups did not differ significantly. However, IBD patients were more frequently edentulous in every sextant (35).

### Risk of developing inflammatory bowel disease in periodontitis group compared to IBD-free patients (PECO 2)

Due to the low number of eligible studies, we can conclude that there are tendencies that should form the subject of future investigations. However, both eligible articles found that patients with UC – but not CD – tend to have periodontitis more frequently than the control population. The reason for this could be the slightly different pathogenesis of the two types of IBD (62).

### Possible reasons for the positive association

Both periodontitis and IBD are multifactorial diseases, and their pathomechanisms have been the focus of recent investigations. (63) In their pathogenesis, a pathological immune-inflammatory response, genetic susceptibility, dysbiosis, and environmental factors play key roles, and they have an impact on each other.

### Common behavioral and environmental factors

It is known that smoking is one of the most harmful environmental factors that increases the risk of periodontitis. It has also been shown in multiple studies that smoking is a significant effect modifier. Brito et al. evaluated the periodontal outcomes of former and current smokers separately from those of non-smokers. They found that the prevalence of periodontitis was higher among smokers with UC than among UC-free smokers. However, there was no difference in the prevalence among non-smoker controls and the non-smoker population with IBD (27). Also, Vavricka et al. found in their study that non-smoking decreased the risk of periodontitis in the case of IBD patients as well, while previous smoking also increased the risk of periodontitis (37). It should also be mentioned that smoking increases the risk of CD (and periodontitis) but this is not completely true for UC, underlying the slightly different pathogenesis of the two inflammatory bowel diseases (64). Another significant risk factor for developing periodontitis is poor oral hygiene. IBD patients are likely to have oral symptoms, like ulcers, aphthous stomatitis, cobblestoning, tag-like lesions, mucogingivis, etc., which may cause pain and make chewing and swallowing difficult and painful (65). A major limitation of most studies is that the researchers did not observe the oral health status of the participants; thus, the population is not standardized. Moreover, if participants’ oral hygiene status was included, it was recorded in very different ways (e.g., the frequency of tooth brushing or the use of dental floss or BOP, PI, etc.). Therefore, we could not compare those studies by subgroups in our meta-analysis.

### Common immune-inflammatory pathways

Altered immune-inflammatory responses also play a key role in both IBD and periodontitis. In their pathogenesis, both the innate and the adaptive immunity are distorted. Several different alterations in the immune-inflammatory response and the mistake in pathogen recognition lead to an imbalance of the protective and inflammatory response, and therefore maintain chronic inflammation (64). However, there are several similar pathways suggesting that the diseases have similar backgrounds, although there are also clear differences between the diseases. It has been shown that in the pathogenesis of CD, Th1 cells play a key role, and an excessive production of IL-12 and IFN-γ can be observed. However, ulcerative colitis is mediated by Th2 cells, and excessive IL-13 production can be observed (66). It has been found that the concentration of IL-18 in the serum is higher in the case of periodontitis patients with CD or UC compared to controls (67). Chi et al. found in their study that steroids used to treat IBD have a protective effect against periodontitis, which supports the hypothesis that both diseases might share common altered immune-inflammatory mechanisms (28). However, they found that other medications taken by IBD groups – 5-ASA products, azathioprine and other immuno-suppressants – had no significant effect on periodontitis. Also, it is one of the most significant limitations of all the studies that the IBD population is very heterogenous regarding the IBD-related medication, but medication could be a very serious effect modifier, causing a bias. It is clear, that the altered immune-inflammatory response is a significant factor in the pathogenesis of the diseases. However, there is currently insufficient data to fully understand the pathogenesis of the disease and so we cannot determine whether it is responsible for the association between IBD and periodontitis or not.

### Common bacterial changes

In the pathogenesis of both IBD and periodontitis, the inflammatory response to different bacteria plays a crucial role. Alterations of the microbiome, which is the dysbiosis is the key factor in the pathogenesis. The development of periodontitis is accompanied by a shift in the subgingival communities from mainly gram-positive aerobic species to gram-negative anaerobic species, triggering an uncontrolled immune-inflammatory response and leading to tissue destruction (68). One of the key pathogens in periodontitis is *Porphyromonas gingivalis*, which has several virulence factors and is responsible for causing dysbiosis and tissue destruction (69). It has been observed that swallowed *P. gingivalis* causes alterations to the gut microbiota, which increases the epithelial permeability of the gut and causes endotoxemia, leading to systemic inflammation (70).

### Common genetic pathways

Genetic predisposition is also instrumental in facilitating the development of both IBD and periodontitis. Positive family history can increase the risk of developing these diseases. There is no exact genetic mutation known that is responsible for these diseases, but some mutations are known to be associated with the diseases. A specific mutation in the nucleotide binding oligomerization domain 2/caspase recruitment domain 15 (NOD2/CARD15) gene is strongly associated with CD (71). Other studies showed that IL-23 R mutation contributes to ATG16L mutation, causing an altered inflammatory response in the case of CD (72). Family accumulation can also be observed in periodontitis. However, further studies are needed to understand the genetic background of these diseases.

### Strengths and limitations

To the best of our knowledge, this is the first meta-analysis to investigate the association between periodontitis and inflammatory bowel diseases separately in both directions, evaluating studies with different exposed and control groups in order to determine whether IBD and periodontitis have a bidirectional association. We could utilize 6 studies in the meta-analysis, and 14 in the systematic review.

Regarding the strengths of our analysis, we followed our protocol, which was registered in advance. A rigorous methodology was applied.

According to the risk of bias assessment, none of the studies included in our statistical evaluation was of low methodological quality. The certainty of evidence was moderate, but the only reason for not ranking high in quality was the design of studies, as using RCTs in this meta-analysis would have been impossible. No other reason was found to reduce the certainty by any domains.

As to the limitations, the definition of periodontitis differed among the studies or was missing altogether. Different screening techniques for PPD, CAL, gingival, and plaque indices were applied in the articles, which prevented making a high-quality statistical evaluation. For the gingival and plaque indices, the small number of articles using the same methods prohibited quantitative synthesis. For PPD and CAL the evaluation was viable, yet the heterogeneity was high; therefore, the results should be considered with caution, and further conclusions should not be made. Only two studies recruiting patients as defined in PECO 2 could be included, with the result that only tendencies could be examined; more studies are needed for reliable statistical results.

Even though the statistical evaluation showed significant link between IBD (both UC and CD) and periodontitis, including RCTs into this meta-analysis was not possible, so we could not prove a cause relationship between the diseases.

### Implication for practice and research

The treatment of IBD remains primarily a gastroenterological issue. Even though IBD is a life-long diagnosis, affected patients are usually unaware of the associated diseases for which they are more susceptible. A systemic inflammation in the body can worsen the stage of the IBD and might induce a flare-up, or inhibit the effectiveness of medication.

Besides, the clinical manifestation of severe periodontitis with active inflammation can be prevented by regular dental check-ups and proper oral hygiene. A multidisciplinary approach should be applied in the treatment of IBD, and dentists should be part of the multidisciplinary team treating IBD patients.

Dentists should routinely ask, if their patient has IBD and therefore take precautionary periodontal measures and gastroenterologists should send their IBD patients to regular dental examinations.

Additional data collection and evaluation is needed to assess the links between IBD and periodontitis more rigorously. An international IBD registry concentrating on the oral manifestations and complications and/or further observational clinical trials with significantly longer follow-up periods might provide additional insight into this topic.

Furthermore, we recommend rigorously following the standardized protocol when measuring different periodontal parameters so that studies can be compared in future meta-analyses.

## Conclusion

The results of our systematic review and meta-analysis confirm that IBD patients have a higher chance of developing periodontitis. However, further studies are needed to investigate the bidirectional association between the diseases. IBD patients are a risk population in dentistry and are more susceptible to periodontitis. Both dentists and gastroenterologists should be aware of this and should emphasize the importance of prevention even more than in the healthy population.

Both diseases are multifactorial, and neither pathogenesis is fully understood. Investigating the association and finding the reason for the positive association could help us understand the nature of these diseases. We might find common immunological or genetic pathways, or identify common behavioral or environmental risk factors.

## Data availability statement

The original contributions presented in this study are included in the article/Supplementary material, further inquiries can be directed to the corresponding author.

## Author contributions

ZD: conceptualization, project administration, methodology, formal analysis, and writing – original draft. EU: conceptualization, formal analysis, visualization, and writing – review and editing. BS: investigation, visualization, validation, and writing – review and editing. PHeg, MC, GV, and ON: conceptualization, methodology, supervision, and writing – review and editing. FD: conceptualization, formal analysis, and writing – review and editing. PHer and BK: supervision and writing – review and editing. All authors provided critical conceptual input and approved the final version of the article, certify that they have participated sufficiently in the work to take public responsibility for its content, including participation in the concept, design, analysis, writing, or revision of the manuscript.

## References

[1] Global Burden of Disease Collaborative Network. *Global Burden of Disease Study 2019.* (2020). Available online at: http://ghdx.healthdata.org/gbd-results-tool (accessed on June 13, 2022).

[2] Chapple ILC Mealey BL Van Dyke TE Bartold PM Dommisch H Eickholz P Periodontal health and gingival diseases and conditions on an intact and a reduced periodontium: consensus report of workgroup 1 of the 2017 world workshop on the classification of periodontal and peri-implant diseases and conditions. *J Periodontol.* (2018) 89(Suppl 1.):S74–84. 10.1002/JPER.17-0719 29926944

[3] Papapanou PN. Periodontal diseases: epidemiology. *Ann Periodontol.* (1996) 1:1–36. 10.1902/annals.1996.1.1.1 9118256

[4] Page RC Offenbacher S Schroeder HE Seymour GJ Kornman KS. Advances in the pathogenesis of periodontitis: summary of developments, clinical implications and future directions. *Periodontol.* (1997) 14:216–48. 10.1111/j.1600-0757.1997.tb00199.x 9567973

[5] Preshaw PM Alba AL Herrera D Jepsen S Konstantinidis A Makrilakis K Periodontitis and diabetes: a two-way relationship. *Diabetologia.* (2012) 55:21–31. 10.1007/s00125-011-2342-y 22057194 PMC3228943

[6] Kinane DF Marshall GJ. Periodontal manifestations of systemic disease. *Aust Dent J.* (2001) 46:2–12. 10.1111/j.1834-7819.2001.tb00267.x 11355236

[7] Papapanou PN Sanz M Buduneli N Dietrich T Feres M Fine DH Periodontitis: consensus report of workgroup 2 of the 2017 world workshop on the classification of periodontal and peri-implant diseases and conditions. *J Periodontol.* (2018) 89(Suppl 1.):S173–82. 10.1002/JPER.17-0721 29926951

[8] Ide M Papapanou PN. Epidemiology of association between maternal periodontal disease and adverse pregnancy outcomes–systematic review. *J Periodontol.* (2013) 84(4 Suppl.):S181–94. 10.1902/jop.2013.134009 23631578

[9] Humphrey LL Fu R Buckley DI Freeman M Helfand M. Periodontal disease and coronary heart disease incidence: a systematic review and meta-analysis. *J Gen Intern Med.* (2008) 23:2079–86. 10.1007/s11606-008-0787-6 18807098 PMC2596495

[10] Sanz M Marco Del Castillo A Jepsen S Gonzalez-Juanatey JR D’Aiuto FP Bouchard P Periodontitis and cardiovascular diseases: consensus report. *J Clin Periodontol.* (2020) 47:268–88. 10.1111/jcpe.13189 32011025 PMC7027895

[11] Bartold PM Lopez-Oliva I. Periodontitis and rheumatoid arthritis: an update 2012-2017. *Eriodontol.* (2020) 83:189–212. 10.1111/prd.12300 32385878

[12] Ide M Harris M Stevens A Sussams R Hopkins V Culliford D Periodontitis and cognitive decline in Alzheimer’s disease. *PLoS One.* (2016) 11:e0151081. 10.1371/journal.pone.0151081 26963387 PMC4786266

[13] Moghadam SA Shirzaiy M Risbaf S. The associations between periodontitis and respiratory disease. *J Nepal Health Res Counc.* (2017) 15:1–6. 10.3126/jnhrc.v15i1.18023 28714484

[14] Nwizu N Wactawski-Wende J Genco RJ. Periodontal disease and cancer: epidemiologic studies and possible mechanisms. *Periodontol.* (2020) 83:213–33. 10.1111/prd.12329 32385885 PMC7328760

[15] Schmidt J Weigert M Leuschner C Hartmann H Raddatz D Haak R Active matrix metalloproteinase-8 and periodontal bacteria-interlink between periodontitis and inflammatory bowel disease? *J Periodontol.* (2018) 89:699–707. 10.1002/JPER.17-0486 29574823

[16] Yu HC Chen TP Chang YC. Inflammatory bowel disease as a risk factor for periodontitis under taiwanese national health insurance research database. *J Dent Sci.* (2018) 13:242–7. 10.1016/j.jds.2018.03.004 30895127 PMC6388870

[17] Zhang L Gao X Zhou J Chen S Zhang J Zhang Y Increased risks of dental caries and periodontal disease in Chinese patients with inflammatory bowel disease. *Int Dent J.* (2020) 70:227–36. 10.1111/idj.12542 31998967 PMC9379173

[18] Sairenji T Collins KL Evans DV. An update on inflammatory bowel disease. *Prim Care.* (2017) 44:673–92. 10.1016/j.pop.2017.07.010 29132528

[19] Molodecky NA Soon IS Rabi DM Ghali WA Ferris M Chernoff G Increasing incidence and prevalence of the inflammatory bowel diseases with time, based on systematic review. *Gastroenterology.* (2012) 142:46–54.e42. 10.1053/j.gastro.2011.10.001 22001864

[20] Feuerstein JD Cheifetz AS. Crohn disease: epidemiology, diagnosis, and management. *Mayo Clin Proc.* (2017) 92:1088–103. 10.1016/j.mayocp.2017.04.010 28601423

[21] Feuerstein JD Cheifetz AS. Ulcerative colitis: epidemiology, diagnosis, and management. *Mayo Clin Proc.* (2014) 89:1553–63. 10.1016/j.mayocp.2014.07.002 25199861

[22] Zhang YZ Li YY. Inflammatory bowel disease: pathogenesis. *World J Gastroenterol.* (2014) 20:91–9. 10.3748/wjg.v20.i1.91 24415861 PMC3886036

[23] Hajishengallis G Chavakis T. Local and systemic mechanisms linking periodontal disease and inflammatory comorbidities. *Nat Rev Immunol.* (2021) 21:426–40. 10.1038/s41577-020-00488-6 33510490 PMC7841384

[24] Vavricka SR Schoepfer A Scharl M Lakatos PL Navarini A Rogler G. Extraintestinal manifestations of inflammatory bowel disease. *Inflamm Bowel Dis.* (2015) 21:1982–92. 10.1097/MIB.0000000000000392 26154136 PMC4511685

[25] Tan CX Brand HS de Boer NK Forouzanfar T. Gastrointestinal diseases and their oro-dental manifestations: part 1: Crohn’s disease. *Br Dent J.* (2016) 221:794–9. 10.1038/sj.bdj.2016.954 27982000

[26] Tan CX Brand HS de Boer NK Forouzanfar T. Gastrointestinal diseases and their oro-dental manifestations: part 2: Ulcerative colitis. *Br Dent J.* (2017) 222:53–7. 10.1038/sj.bdj.2017.37 28084352

[27] Brito F de Barros FC Zaltman C Carvalho AT Carneiro AJ Fischer RG Prevalence of periodontitis and DMFT index in patients with Crohn’s disease and ulcerative colitis. *J Clin Periodontol.* (2008) 35:555–60. 10.1111/j.1600-051X.2008.01231.x 18400026

[28] Chi YC Chen JL Wang LH Chang K Wu CL Lin SY Increased risk of periodontitis among patients with Crohn’s disease: a population-based matched-cohort study. *Int J Colorectal Dis.* (2018) 33:1437–44. 10.1007/s00384-018-3117-4 30003361

[29] Grössner-Schreiber B Fetter T Hedderich J Kocher T Schreiber S Jepsen S. Prevalence of dental caries and periodontal disease in patients with inflammatory bowel disease: a case-control study. *J Clin Periodontol.* (2006) 33:478–84. 10.1111/j.1600-051X.2006.00942.x 16820035

[30] Habashneh RA Khader YS Alhumouz MK Jadallah K Ajlouni Y. The association between inflammatory bowel disease and periodontitis among Jordanians: a case-control study. *J Periodontal Res.* (2012) 47:293–8. 10.1111/j.1600-0765.2011.01431.x 22050539

[31] Kang EA Chun J Kim JH Han K Soh H Park S Periodontitis combined with smoking increases risk of the ulcerative colitis: a national cohort study. *World J Gastroenterol.* (2020) 26:5661–72. 10.3748/wjg.v26.i37.5661 33088159 PMC7545388

[32] Koutsochristou V Zellos A Dimakou K Panayotou I Siahanidou S Roma-Giannikou E Dental caries and periodontal disease in children and adolescents with inflammatory bowel disease: a case-control study. *Inflamm Bowel Dis.* (2015) 21:1839–46. 10.1097/MIB.0000000000000452 25985243

[33] Lin CY Tseng KS Liu JM Chuang HC Lien CH Chen YC Increased risk of ulcerative colitis in patients with periodontal disease: a nationwide population-based cohort study. *Int J Environ Res Public Health.* (2018) 15:2602. 10.3390/ijerph15112602 30469385 PMC6265883

[34] Slebioda Z Szponar E Linke K. Comparative analysis of the oral cavity status in patients with Crohn’s disease and ulcerative colitis. *J Stomatol.* (2011) 64:212–24.

[35] Tan CXW Brand HS Kalender B De Boer NKH Forouzanfar T de Visscher J. Dental and periodontal disease in patients with inflammatory bowel disease. *Clin Oral Investig.* (2021) 25:5273–80. 10.1007/s00784-021-03835-6 33619633 PMC8370899

[36] Zervou F Gikas A Merikas E Peros G Sklavaina M Loukopoulos J Oral manifestations of patients with inflammatory bowel disease. *Ann Gastroenterol.* (2004) 17:395–401.

[37] Vavricka SR Manser CN Hediger S Vögelin M Scharl M Biedermann L Periodontitis and gingivitis in inflammatory bowel disease: a case-control study. *Inflamm Bowel Dis.* (2013) 19:2768–77. 10.1097/01.MIB.0000438356.84263.3b 24216685

[38] Zhang Y Qiao D Chen R Zhu F Gong J Yan F. The association between periodontitis and inflammatory bowel disease: a systematic review and meta-analysis. *Biomed Res Int.* (2021) 2021:6692420. 10.1155/2021/6692420 33778080 PMC7981176

[39] Papageorgiou SN Hagner M Nogueira AV Franke A Jäger A Deschner J. Inflammatory bowel disease and oral health: systematic review and a meta-analysis. *J Clin Periodontol.* (2017) 44:382–93. 10.1111/jcpe.12698 28117909

[40] Lorenzo-Pouso AI Castelo-Baz P Rodriguez-Zorrilla S Pérez-Sayáns M Vega P. Association between periodontal disease and inflammatory bowel disease: a systematic review and meta-analysis. *Acta Odontol Scand.* (2021) 79:344–53. 10.1080/00016357.2020.1859132 33370548

[41] She YY Kong XB Ge YP Liu ZY Chen JY Jiang JW Periodontitis and inflammatory bowel disease: a meta-analysis. *BMC Oral Health.* (2020) 20:67. 10.1186/s12903-020-1053-5 32164696 PMC7069057

[42] Page MJ McKenzie JE Bossuyt PM Boutron I Hoffmann TC Mulrow CD The prisma 2020 statement: an updated guideline for reporting systematic reviews. *BMJ.* (2021) 372:n71. 10.1136/bmj.n71 33782057 PMC8005924

[43] Higgins JPT Thomas J Chandler J Cumpston M Li T Page MJ (eds). *Cochrane handbook for systematic reviews of interventions version 6.3 (updated February 2022).* Cochrane (2022). Available online at: www.training.cochrane.org/handbook

[44] McHugh ML. Interrater reliability: the kappa statistic. *Biochem Med.* (2012) 22:276–82. 10.11613/BM.2012.031 PMC3900052 23092060

[45] GA Wells BS O’Connell D Peterson J Welch V Losos M Tugwell P. *The newcastle-ottawa scale (NOS) for assessing the quality of nonrandomised studies in meta-analyses.* (2021). Available online at: http://www.ohri.ca/programs/clinical_epidemiology/oxford.asp

[46] Mantel N Haenszel W. Statistical aspects of the analysis of data from retrospective studies of disease. *J Natl Cancer Inst.* (1959) 22:719–48. 13655060

[47] Robins J Greenland S Breslow NE. A general estimator for the variance of the Mantel-Haenszel odds ratio. *Am J Epidemiol.* (1986) 124:719–23. 10.1093/oxfordjournals.aje.a114447 3766505

[48] Thompson SG Turner RM Warn DE. Multilevel models for meta-analysis, and their application to absolute risk differences. *Stat Methods Med Res.* (2001) 10:375–92. 10.1191/096228001682157616 11763548

[49] Cooper H Hedges LV Valentine JC. *The Handbook of Research Synthesis and Meta-Analysis.* 2nd ed. New York: Russel Sage Foundation (2009).

[50] Sweeting MJ Sutton AJ Lambert PC. What to add to nothing? Use and avoidance of continuity corrections in meta-analysis of sparse data. *Stat Med.* (2004) 23:1351–75. 10.1002/sim.1761 15116347

[51] Knapp G Hartung J. Improved tests for a random effects meta-regression with a single covariate. *Stat Med.* (2003) 22:2693–710. 10.1002/sim.1482 12939780

[52] IntHout J Ioannidis JP Borm GF. The Hartung-Knapp-Sidik-Jonkman method for random effects meta-analysis is straightforward and considerably outperforms the standard DerSimonian-Laird method. *BMC Med Res Methodol.* (2014) 14:25. 10.1186/1471-2288-14-25 24548571 PMC4015721

[53] Paule RC Mandel J. Consensus values, regressions, and weighting factors. *J Res Natl Inst Stand Technol.* (1989) 94:197–203. 10.6028/jres.094.020 28053410 PMC4943748

[54] Veroniki AA Jackson D Viechtbauer W Bender R Bowden J Knapp G Methods to estimate the between-study variance and its uncertainty in meta-analysis. *Res Synth Methods.* (2016) 7:55–79. 10.1002/jrsm.1164 26332144 PMC4950030

[55] Higgins JP Thompson SG. Quantifying heterogeneity in a meta-analysis. *Stat Med.* (2002) 21:1539–58. 10.1002/sim.1186 12111919

[56] Harrer M Cuijpers P Furukawa TA Ebert DD. *Doing Meta-Analysis With R: A Hands-On Guide.* 1st ed. Boca Raton, FL: Chapman & Hall/CRC (2021). 10.1201/9781003107347

[57] Viechtbauer W Cheung MW. Outlier and influence diagnostics for meta-analysis. *Res Synth Methods.* (2010) 1:112–25. 10.1002/jrsm.11 26061377

[58] Harbord RM Egger M Sterne JA. A modified test for small-study effects in meta-analyses of controlled trials with binary endpoints. *Stat Med.* (2006) 25:3443–57. 10.1002/sim.2380 16345038

[59] Sterne JA Sutton AJ Ioannidis JP Terrin N Jones DR Lau J Recommendations for examining and interpreting funnel plot asymmetry in meta-analyses of randomised controlled trials. *BMJ.* (2011) 343:d4002. 10.1136/bmj.d4002 21784880

[60] Schumemann H Brożek J Guyatt G Oxman A. *GRADE handbook. Grading of recommendations assessment, development and evaluation, grade working group*. (2013). Available online at: https://gdt.gradepro.org/app/handbook/handbook.html

[61] Zhang L Li X Yan H Huang L. Salivary matrix metalloproteinase (MMP)-8 as a biomarker for periodontitis: a prisma-compliant systematic review and meta-analysis. *Medicine.* (2018) 97:e9642. 10.1097/MD.0000000000009642 29504999 PMC5779768

[62] Sartor RB. Mechanisms of disease: pathogenesis of Crohn’s disease and ulcerative colitis. *Nat Clin Pract Gastroenterol Hepatol.* (2006) 3:390–407. 10.1038/ncpgasthep0528 16819502

[63] Lira-Junior R Figueredo CM. Periodontal and inflammatory bowel diseases: is there evidence of complex pathogenic interactions? *World J Gastroenterol.* (2016) 22:7963–72. 10.3748/wjg.v22.i35.7963 27672291 PMC5028810

[64] Indriolo A Greco S Ravelli P Fagiuoli S. What can we learn about biofilm/host interactions from the study of inflammatory bowel disease. *J Clin Periodontol.* (2011) 38(Suppl 11.):36–43. 10.1111/j.1600-051X.2010.01680.x 21323702

[65] Ribaldone DG Brigo S Mangia M Saracco GM Astegiano M Pellicano R. Oral manifestations of inflammatory bowel disease and the role of non-invasive surrogate markers of disease activity. *Medicines.* (2020) 7:33. 10.3390/medicines7060033 32560118 PMC7345678

[66] Bouma G Strober W. The immunological and genetic basis of inflammatory bowel disease. *Nat Rev Immunol.* (2003) 3:521–33. 10.1038/nri1132 12876555

[67] Figueredo CM Brito F Barros FC Menegat JS Pedreira RR Fischer RG Expression of cytokines in the gingival crevicular fluid and serum from patients with inflammatory bowel disease and untreated chronic periodontitis. *J Periodontal Res.* (2011) 46:141–6. 10.1111/j.1600-0765.2010.01303.x 20701671

[68] Curtis MA Diaz PI Van Dyke TE. The role of the microbiota in periodontal disease. *Periodontol.* (2020) 83:14–25. 10.1111/prd.12296 32385883

[69] Khan SA Kong EF Meiller TF Jabra-Rizk MA. Periodontal diseases: bug induced, host promoted. *PLoS Pathog.* (2015) 11:e1004952. 10.1371/journal.ppat.1004952 26226364 PMC4520614

[70] Hajishengallis G. Periodontitis: from microbial immune subversion to systemic inflammation. *Nat Rev Immunol.* (2015) 15:30–44. 10.1038/nri3785 25534621 PMC4276050

[71] Hugot JP Chamaillard M Zouali H Lesage S Cézard JP Belaiche J Association of NOD2 leucine-rich repeat variants with susceptibility to Crohn’s disease. *Nature.* (2001) 411:599–603. 10.1038/35079107 11385576

[72] Rioux JD Xavier RJ Taylor KD Silverberg MS Goyette P Huett A Genome-wide association study identifies new susceptibility loci for Crohn disease and implicates autophagy in disease pathogenesis. *Nat Genet.* (2007) 39:596–604. 10.1038/ng2032 17435756 PMC2757939

