# Supplementary material for: Patients with inflammatory bowel disease have a higher chance of developing periodontitis: A systematic review and meta-analysis
Source: Front Med (Lausanne). 2022 Nov 8;9:1020126. doi: 10.3389/fmed.2022.1020126 (PMC9679143; doi:10.3389/fmed.2022.1020126)
Supplement: Supplementary file 1 [file Data_Sheet_1.pdf]

Supplementary material

Supplementary Figure 1

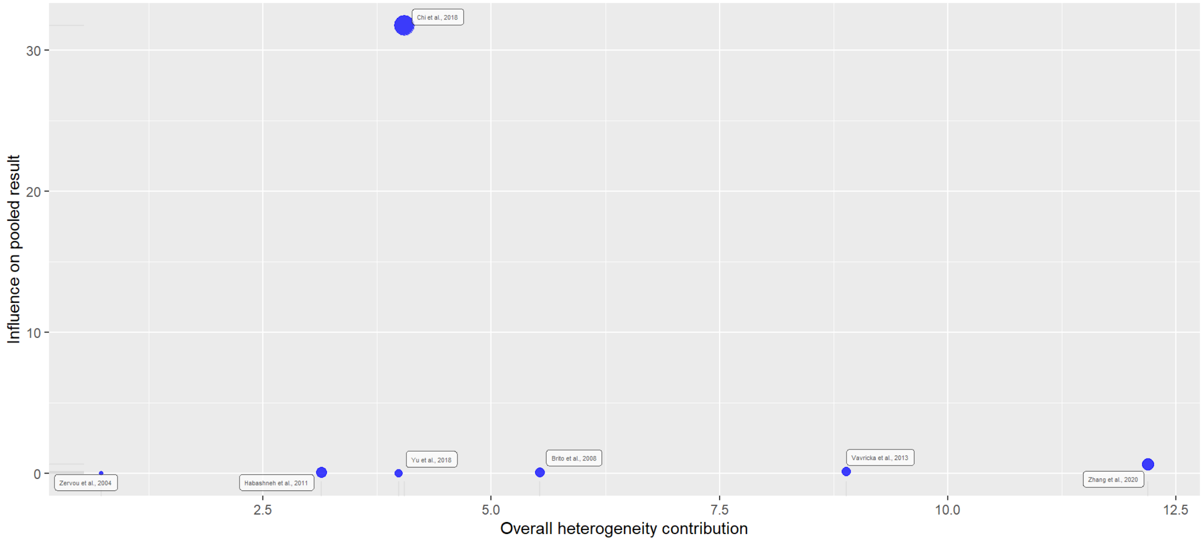

Supplementary Figure 1. Baujat plot, showing the study by Chi et al. is an outlier

Supplementary Figure 2 – IBD funnel plot

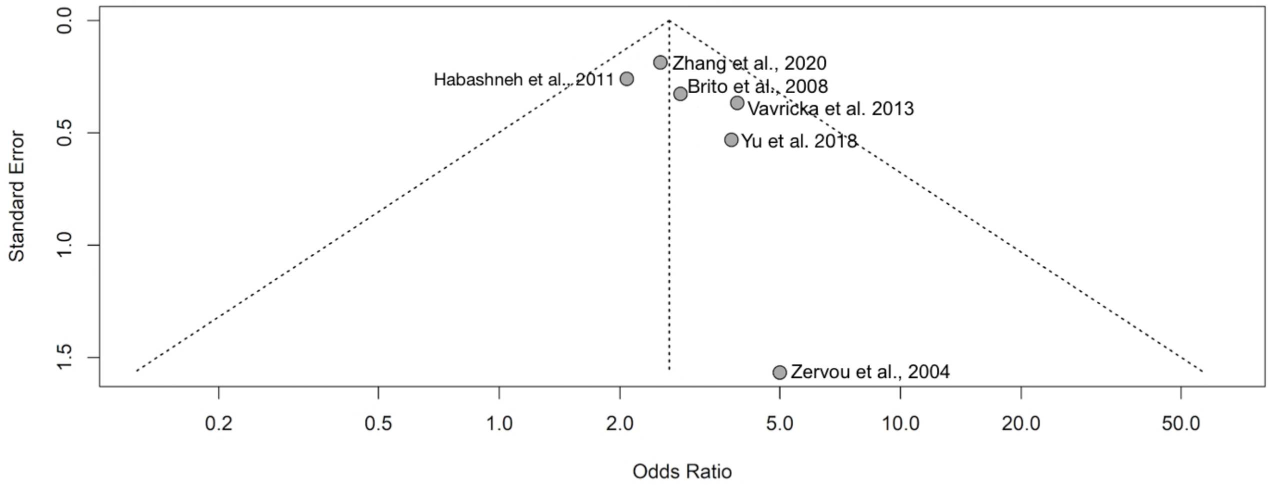

Supplementary Figure 2. Funnel plot for odds ratio in inflammatory bowel disease (IBD) group

Supplementary Figure 3

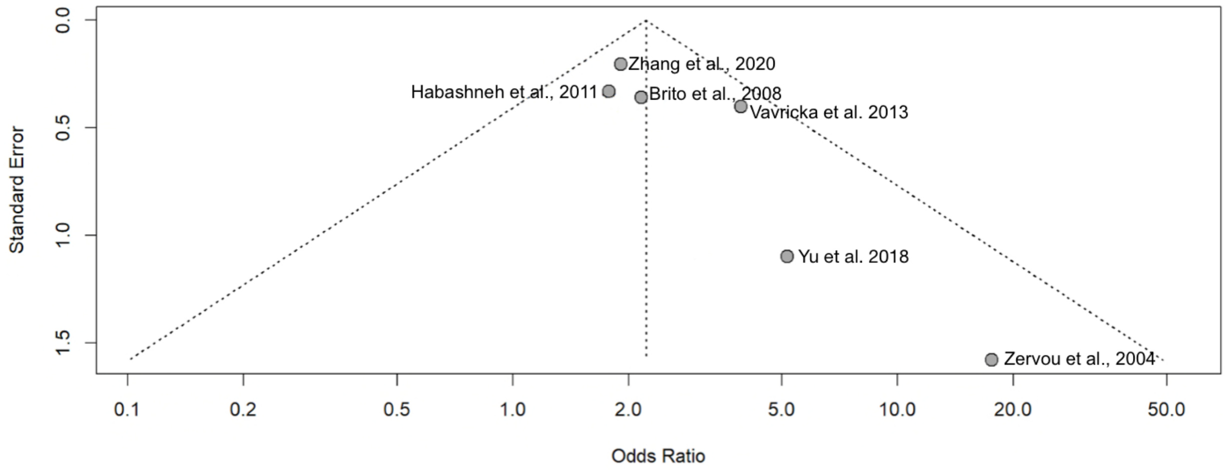

Supplementary Figure 3. Funnel plot for odds ratio in Crohn's disease (CD) group

Supplementary Figure 4

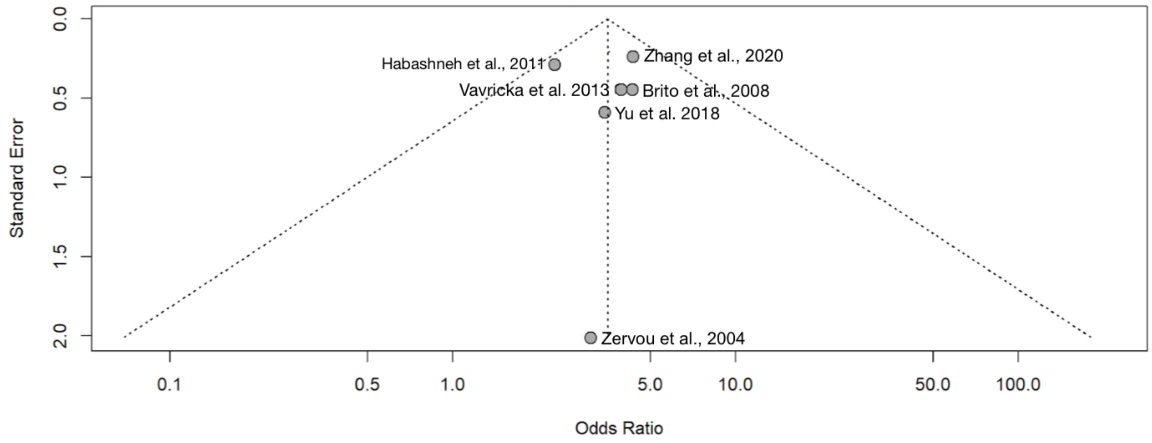

Supplementary Figure 4. Funnel plot for odds ratio in ulcerative colitis (UC) group

Supplementary Figure 2, 3 and 4: Publication bias was evaluated by funnel plot and Egger's test. However, the meta-analysis contains few studies therefore Egger's test may lack the statistical power to detect bias or it could give false "positive" result, so publication bias could not be analyzed.

Supplementary Figure 5

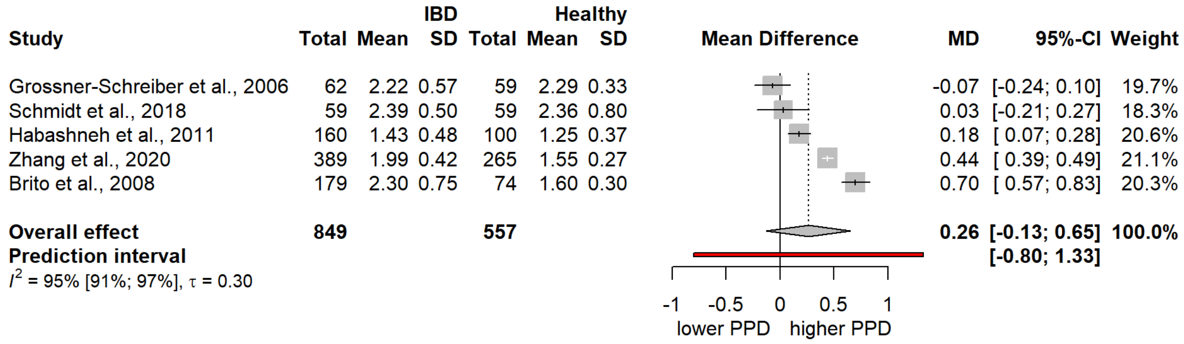

Supplementary Figure 5. Difference in mean Probing Pocket Depth (PPD) results between inflammatory bowel disease (IBD) and IBD-free patients
Mean difference (MD), and confidence interval (CI)

Supplementary Figure 6

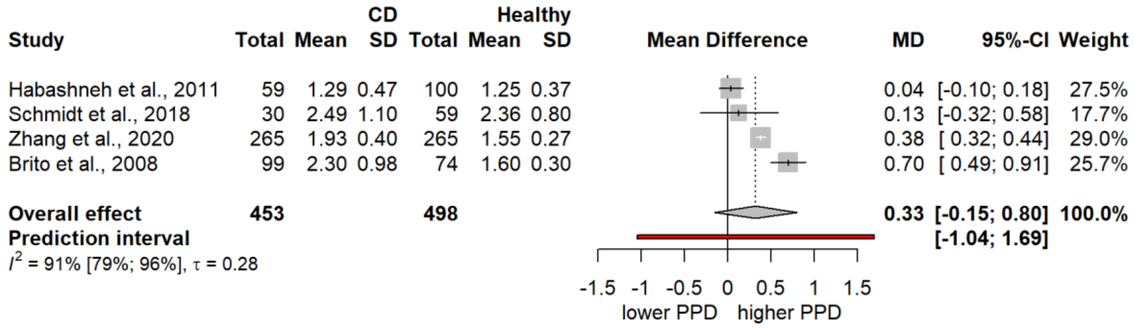

Supplementary Figure 6. Difference in mean Probing Pocket Depth (PPD) results between Crohn’s disease (CD) and inflammatory bowel disease (IBD)-free patients
Mean difference (MD), and confidence interval (CI)

Supplementary Figure 7

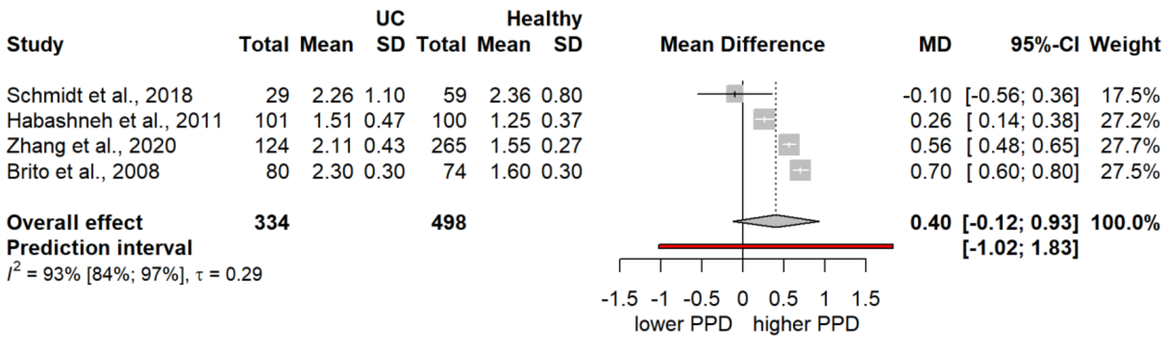

Supplementary Figure 7. Difference in mean Probing Pocket Depth (PPD) results between ulcerative colitis (UC) and inflammatory bowel disease (IBD)-free patients Mean difference (MD), and confidence interval (CI)

Supplementary Figure 8

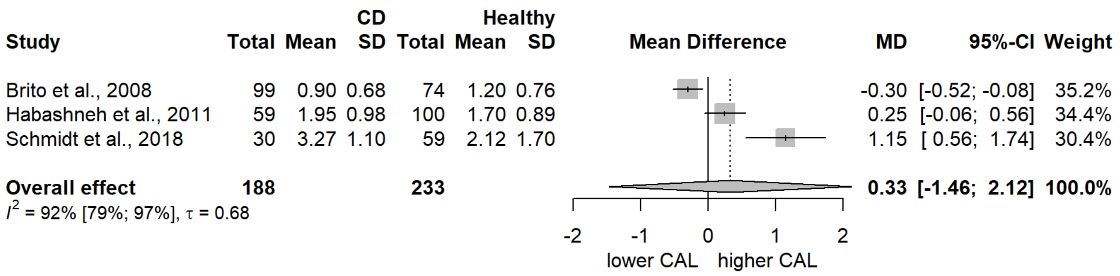

Supplementary Figure 8. Difference in Clinical Attachment Loss (CAL) results between Crohn’s disease (CD)-patients and inflammatory bowel disease (IBD)-free patients Mean difference (MD), and confidence interval (CI)

Supplementary Figure 9

Study	UC Total	UC Mean	UC SD	Healthy Total	Healthy Mean	Healthy SD	MD	95%-CI	Weight
Brito et al., 2008	80	1.30	1.06	74	1.20	0.76	0.10	[-0.19; 0.39]	36.7%
Habashneh et al., 2011	101	2.36	1.13	100	1.70	0.89	0.66	[0.38; 0.94]	36.8%
Schmidt et al., 2018	29	3.39	1.50	59	2.12	1.70	1.27	[0.56; 1.98]	26.5%
**Overall effect**	**210**			**233**			**0.62**	**[0.00; 1.24]**	**100.0%**

I^2 = 85% [55%; 95%], τ = 0.50

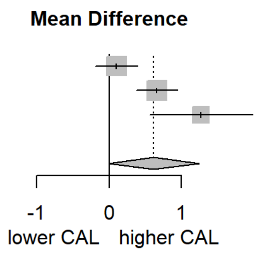

Supplementary Figure 9. Difference in Clinical Attachment Loss (CAL) results between ulcerative colitis (UC)-patients and inflammatory bowel disease (IBD)-free patients Mean difference (MD), and confidence interval (CI)

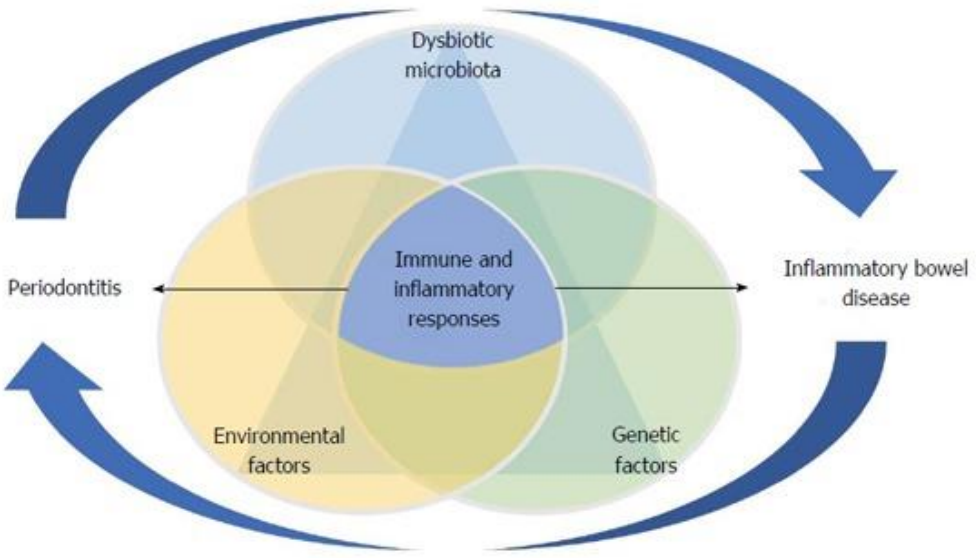

Supplementary Figure 10. – Possible common genetical pathways between IBD and periodontitis, figure from Lira-Junior et. al. 2016 [1]

PECO 1

Supplementary Table 1. PECO 1

**P (Population)**	Human beings, regardless of age, sex (exclusion: edentolous patients)
**E (Exposure)**	Diagnosis of inflammatory bowel disease (including Crohn's disease or ulcerative colitis) Regardless of type of IBD, treatment for IBD, time of IBD diagnosis.
**C (Control)**	Absence of inflammatory bowel disease
**O (Outcome)**	**Main:** Prevalence of periodontitis. The definition of periodontitis used by the authors of the study is attached<br>**Secondary:** any clinical periodontal parameters examined in the study (PPD, GR, CAL, BOP, PI, GI, CPITN, etc.)

PECO 2

Supplementary Table 2. PECO 2

**P**	Human beings
**E**	Diagnosis of periodontitis accompanied by the definition of the disease given by the authors
**C**	Absence of periodontitis
**O**	Prevalence of inflammatory bowel disease. (Prevalence of Crohn's disease. Prevalence of ulcerative colitis.)

Supplementary Table 3. Hits in databases with the used search strategy

**Database**	**Hits up to October 26, 2021**
MEDLINE	1,087
Embase	524
CENTRAL	104

During the systematic search the following search key was used:
(periodontitis OR chronic periodontitis OR periodontal OR periodontal disease) AND (inflammatory bowel disease OR Crohn* OR ulcerative colitis OR uc OR cd OR ibd)

The same search query was used in all three databases.

Hits are shown up to October 26, 2021.

Supplementary Table 4. Risk of bias assessment for case-control studies

Case-control studies

	Selection				Comparability	Exposure			All
	Is the case definition adequate?	Representativeness of the cases	Selection of controls	Definition of controls	Comparability of cases and controls on the basis of the design or analysis	Ascertainment of exposure	Same method of ascertainment for cases and controls	Non-Response rate	
Brito et al. 2008	✸	✸		✸	✸	✸	✸	✸	**7**
Grössner-Schreiber et al. 2006	✸	✸	✸	✸	✸✸	✸	✸	✸	**9**
Habashneh et al. 2011	✸	✸		✸	✸✸	✸	✸	✸	**8**
Koutschristou et al. 2015	✸	✸	✸	✸	✸	✸	✸	✸	**9**
Zervou et al. 2004	✸	✸		✸	✸✸	✸	✸	✸	**8**
Vavricka et al. 2013	✸	✸		✸	✸	✸	✸		**6**
Slebioda et al. 2011	✸	✸	✸	✸	✸	✸	✸	✸	**7**
Zhang et al. 2020	✸	✸		✸	✸✸	✸	✸	✸	**8**
Tan et al. 2021		✸	✸	✸	✸✸		✸		**6**
Schmidt et al. 2018		✸		✸	✸✸	✸	✸	✸	**7**

Supplementary Table 5. Risk of bias assessment for cohort studies

Cohort studies

	Selection				Comparability	Outcome			All
	Representativeness of the exposed cohort	Selection of the non-exposed cohort	Ascertainment of exposure	Demonstration that outcome of interest was not present at start of study	Comparability of cohorts on the basis of the design or analysis	Assessment of outcome	Was follow-up long enough for outcomes to occur?	Adequacy of follow-up of cohorts	
Yu et al. 2018	✸	✸	✸	✸	✸✸	✸	✸	✸	**9**
Chi et al. 2018	✸	✸	✸	✸	✸✸	✸	✸	✸	**9**
Kang et al. 2020	✸	✸	✸	✸	✸	✸	✸	✸	**8**
Lin et al. 2018	✸	✸	✸	✸	✸✸	✸	✸	✸	**9**

Newcastle-Ottawa Scale: A study is judged by three main perspectives: the selection of study groups, the comparability of groups and the ascertainment of the exposure. [3] The lowest

quality study gets 0, the highest gets 9 stars. Studies under 5 stars are considered low, above 5 they are considered moderate or high quality studies.

Supplementary Table 6. Certainty assessment

**Outcome**	**Participants (Number of studies)**	**Risk of bias**	**Inconsistency**	**Indirectness**	**Imprecision**	**Publication bias**	**Large effect**	**Dose response**	**Opposing plausible residual bias and confounding (upgrading)**	**Certainty**
**OR in IBD**	6	Not serious	Not serious No heterogeneity, no reason to downgrade	Not serious Directly relevant, no reason to downgrade	Not serious Adequate sample, no reason to downgrade	Due to the small number of studies formal assessment of reporting bias is not possible, no reason to downgrade	Rated up by one level for large effect	No reason to upgrade	No reason to upgrade	⊕⊕⊕◯ **Moderate**
**OR in CD**	6	Not serious	Not serious Little heterogeneity, no reason to downgrade	Not serious Directly relevant, no reason to downgrade	Not serious Adequate sample, no reason to downgrade	Due to the small number of studies formal assessment of reporting bias is not possible, no reason to downgrade	Rated up by one level for large effect	No reason to upgrade	No reason to upgrade	⊕⊕⊕◯ **Moderate**
**OR in UC**	6	Not serious	Not serious No heterogeneity, no reason to downgrade	Not serious Directly relevant, no reason to downgrade	Not serious Adequate sample, no reason to downgrade	Due to the small number of studies formal assessment of reporting bias is not possible, no reason to downgrade	Rated up by one level for large effect	No reason to upgrade	No reason to upgrade	⊕⊕⊕◯ **Moderate**

We could not calculate the assumed risk per 1,000 patients (IBD-free), and the corresponding risk per 1,000 patients (IBD), as the number of exposed and non-exposed groups was not available in one study.

Supplementary Table 7. Data for statistical evaluation: main outcome

study	n_IBD	n_CD	n_UC	n_e_IBD_P	n_e_CD_P	n_e_CU_P	n_e_IBD_non_P	n_e_CD_non_P	n_e_CU_non_P	n_c	n_c_P	n_c_non_P	OR_IBD	OR_IBD_CI_min	OR_IBD_CI_max	OR_CD	OR_CD_CI_min	OR_CD_CI_max	OR_CU	OR_CU_CI_min	OR_CU_CI_max
Zhang et al., 2020	389	265	124	146	83	63	243	182	61	265	51	214	2,52	1,75	3,64	1,91	1,28	2,86	4,33	2,72	6,91
Yu et al., 2018	27	7	20	22	6	16	5	1	4	108	58	50	3,79	1,34	10,75	5,17	0,6	44,43	3,45	1,08	10,99
Chi et al., 2018	6657	6657	0	743	743	0	5914	5914	0	26628	2523	24105	1,2	1,1	1,31	1,2	1,1	1,31	NA	NA	NA
Vavricka et al., 2013	113	69	44	NA	NA	NA	NA	NA	NA	113	NA	NA	3,92	1,91	8,05	3,91	1,78	8,57	3,94	1,64	9,46
Habashneh et al., 2011	160	59	101	93	32	61	67	27	40	100	40	60	2,08	1,25	3,46	1,78	0,93	3,41	2,29	1,3	4,02
Brito et al., 2008	179	99	80	153	81	72	26	18	8	74	50	24	2,83	1,49	5,36	2,16	1,07	4,37	4,32	1,8	10,39
Zervou et al., 2004	30	15	15	2	2	0	47	13	15	47	0	47	5	0,23	106,95	17,59	0,8	388,97	3,07	0,06	161,03

Supplementary Table 8. Secondary outcome: Probing Pocket Depth (PPD) results

Article	n_IBD_PPD	mean_IBD_PPD	SD_IBD_PPD	n_CD_PPD	mean_CD_PPD	SD_CD_PPD	n_UC_PPD	mean_UC_PPD	SD_UC_PPD	n_c_PPD	mean_c_PPD	SD_c_PPD
Zhang et al., 2020	389	1.987	0.419	265	1.93	0.403	124	44603	0.428	265	1.547	0.268
Schmidt et al., 2018	59	2.39	0.5	30	2.49	44562	29	44618	44562	59	2.36	0.8
Habashneh et al., 2011	160	1.429	0.48	59	44590	0.47	101	1.51	0.47	100	44586	0.37
Brito et al., 2008	179	44595	0.753	99	44595	0.978	80	44595	0.302	74	44567	0.302
Grossner-Schreiber et al., 2006	62	44614	0.57	46	NA	NA	16	NA	NA	59	2.29	0.33

Supplementary Table 9. Secondary outcome: Clinical Attachment Loss (CAL) results

Study	n_CD	n_CU	n_H	CD_mean	CD_SD	CU_mean	CU_SD	H_mean	H_SD
Schmidt et al., 2018	30	29	59	3.27	1.1	3.39	1.5	2.12	1.7
Habashneh et al., 2011	59	101	100	1.95	0.98	2.36	1.13	1.7	0.89
Brito et al., 2008	99	80	74	0.9	0.68	1.3	1.06	1.2	0.76

Supplementary Table 10. Data for PECO 2

study	n_e	n_c	n_e_IBD	n_c_IBD	n_e_CD	n_c_CD	n_e_CU	n_c_CU
Kang et al., 2020	1092825	8857723	818	6524	126	1300	692	5224
Lin et al., 2018	27041	108149	1036	4356	985	4235	55	137

Appendix 1

OR in IBD vs IBD-free population

A total of six studies were selected for analyses covering a total of 1,605 patients out of which not available (NA) patients have interesting outcomes. On average, the odds ratio (the pooled effect size) of having PD was 2.65. The 95% confidence interval of the odds ratio was 2.09 to 3.36, indicating that the mean effect size in the universe of comparable studies could fall in this range. The CI of pooled effect size does not include the effect size 1, indicating that the mean effect size differs from 1. Therefore, we reject the null hypothesis. We can conclude that on average the odds of having PD in the IBD population are higher than those of the healthy population. The between-study heterogeneity expressed as I^2 value was 0 (95% CI: 0 - 0.75), which tells us that 0% of the variance in observed effects reflects variance in true effects rather than sampling error. The variance of true effects (τ^2) was 0, and the standard deviation of true effects (τ^2) was 0. The prediction interval was 1.87 to 3.75. Based on that we would expect in some 95% of all populations comparable to those in the analysis that the true effect size will fall in this range. [4]

OR in CD vs IBD-free population

A total of six studies were selected for analyses covering a total of 1,221 patients out of which NA patients have the interesting outcomes. On average, the odds ratio (the pooled effect size) of having PD was 2.22. The 95% confidence interval of the odds ratio was 1.49 to 3.31, indicating that the mean effect size in the universe of comparable studies could fall in this range. The CI of pooled effect size does not include the effect size 1, indicating that the mean effect size differs from 1. Therefore, we reject the null hypothesis. We can conclude that on average the odds of having PD in CD population are higher than those of the healthy population. The between-study heterogeneity expressed as I^2 value was 0.05 (95% CI: 0 - 0.76), indicating that 5% of the variance in observed effects reflects variance in true effects rather than sampling error. The variance of true effects (τ^2) was 0.01, and the standard deviation of true effects (τ^2) was 0.1. The prediction interval was 1.32 to 3.73. Based on that we would expect in some 95% of all populations comparable to those in the analysis that the true effect size will fall in this range. [4]

OR in UC vs IBD-free group

A total of six studies were selected for analyses covering a total of 1,091 patients out of which NA patients have the interesting outcomes. On average, the odds ratio (the pooled effect size) of having PD was 3.52. The 95% confidence interval of the odds ratio was 2.56 to 4.83, indicating that the mean effect size in the universe of comparable studies could fall in this range. The CI of pooled effect size does not include the effect size 1, indicating that the mean effect size differs from 1. Therefore, we reject the null hypothesis. We can conclude that on average the odds of having PD in the UC population are higher than those of the healthy population. The between-study heterogeneity expressed as I^2 value was 0 (95% CI: 0 - 0.75), indicating that 0% of the variance in observed effects reflects variance in true effects rather than sampling error. The variance of true effects (τ^2) was 0, and the standard deviation of true effects (τ^2) was 0. The prediction interval was 2.3 to 5.38. Based on that we would expect in some 95% of all populations comparable to those in the analysis that the true effect size will fall in this range. [4]

1. Lira-Junior, R. and C.M. Figueredo, *Periodontal and inflammatory bowel diseases: Is there evidence of complex pathogenic interactions?* World J Gastroenterol, 2016. 22(35): p. 7963-72.
2. Page, M.J., J.E. McKenzie, P.M. Bossuyt, I. Boutron, T.C. Hoffmann, C.D. Mulrow, et al., *The PRISMA 2020 statement: an updated guideline for reporting systematic reviews.* BMJ, 2021. 372: p. n71.
3. GA Wells, B.S., D O'Connell, J Peterson, V Welch, M Losos, P Tugwell, *The Newcastle-Ottawa Scale (NOS) for assessing the quality of nonrandomised studies in meta-analyses.* 2021.
4. Borenstein, M., *Common Mistakes in Meta-Analysis and How to Avoid Them.* 2019, USA: Biostat Inc.https://meta-analysis-books.com/.
